# Effects of systematic data reduction on trend estimation from German registration trials

**DOI:** 10.1007/s00122-023-04266-5

**Published:** 2023-01-23

**Authors:** Jens Hartung, Friedrich Laidig, Hans-Peter Piepho

**Affiliations:** Institut for Crop Science, Biostatistics Unit, University of Hohenheim, Stuttgart, Germany

## Abstract

**Key message:**

VCU trials can provide unbiased estimates of post-breeding trends given that all data is used. Dropping data of genotypes tested for up to two years may result in biased post-breeding trend estimates.

**Abstract:**

Increasing yield trends are seen on-farm in Germany. The increase is based on genetic trend in registered genotypes and changes in agronomic practices and climate. To estimate both genetic and non-genetic trends, historical wheat data from variety trials evaluating a varieties’ value for cultivation und use (VCU) were analyzed. VCU datasets include information on varieties as well as on genotypes that were submitted by breeders and tested in trials but could not make it to registration. Therefore, the population of registered varieties (post-registration population) is a subset of the population of genotypes tested in VCU trials (post-breeding population). To assess post-registration genetic trend, historical VCU trial datasets are often reduced, e.g. to registered varieties only. This kind of drop-out mechanism is statistically informative which affects variance component estimates and which can affect trend estimates. To investigate the effect of this informative drop-out on trend estimates, a simulation study was conducted mimicking the structure of German winter wheat VCU trials. Zero post-breeding trends were simulated. Results showed unbiased estimates of post-breeding trends when using all data. When restricting data to genotypes tested for at least three years, a positive genetic trend of 0.11 dt ha^−1^ year^−1^ and a negative non-genetic trend (− 0.11 dt ha^−1^ year^−1^) were observed. Bias increased with increasing genotype-by-year variance and disappeared with random selection. We simulated single-trait selection, whereas decisions in VCU trials consider multiple traits, so selection intensity per trait is considerably lower. Hence, our results provide an upper bound for the bias expected in practice.

**Supplementary Information:**

The online version contains supplementary material available at 10.1007/s00122-023-04266-5.

## Introduction

In Germany, on-farm crop yield has increased during the last decades (Laidig et al. 2017). This yield increase can be due to improvements in genetics and agronomic practices (Schuster 1997). The long-term genetic trend is due to improvements of newly registered varieties. Non-genetic trends can be due to changes in the ratio of producer-to-input prizes (Peltonen-Sainio et al. 2009) and government regulations that limit the application of fertilizer (DÜV 2020). Additionally, climate change can systematically affect crop yield (DaMatta et al. 2010). It is important for breeders and farmers to dissect genetic und non-genetic effects on yield trends in long-term variety trial data because these two sources determine the overall trend. Farmers require a solid basis to evaluate their decisions on growing newly registered varieties, whereas breeders are interested in measuring their success and planning their future breeding aims (Schuster 1997). Separating genetic and non-genetic components of trend allows prioritizing future research and development to areas with the largest expected progress (Rizzo et al. 2022).

Two types of trials can be used to estimate long-term genetic and non-genetic yield trends in wheat and other crops (Brancourt-Hulmel et al. 2003) for a target region: vintage trials and historical data from trials that evaluate varieties’ value of cultivation and use (VCU) (Fisher et al. 2014; Laidig et al. 2017). The most common approaches are vintage trials or ERA studies (Cooper et al 2020). They are used for a direct comparison of old and modern varieties in the same trials (e.g. Ahrends et al. 2018; Brancourt-Hulmel et al. 2003; Bulman et al. 1993; Curin et al. 2021; Cox et al. 1988; Morgounov et al. 2010; Morrison et al. 2000; Nehe et al. 2019; Ormoli 2015; Perry and D’Antuono 1989; Sanchez-Garcia 2015; Sun et al. 2014). In vintage trials, a limited number of selected varieties spanning a wide range of registration years (usually 10–20 years) are tested for a small number of years (usually two to four years) at a limited number of locations under present environmental conditions. Testing old and new varieties in the same experiment has the advantage that agronomic practices can be set to be the same for all varieties. Therefore, trends seen in vintage trials are directly attributable to genetic trend. For a fairer comparison, however, agronomic conditions such as the amount of fertilizer and growth regulator applied may be varied within the experiment to account for temporal changes in these factors (Brancourt-Hulmel et al. 2003). The idea of vintage trials is to grow varieties under a range of different environmental conditions including conditions they are selected for. This allows estimating the variance due to variety-by-environmental condition interactions. While agronomic management can be adapted to historical conditions, it is not possible to account for changes in climate and pathogenic pressure. Therefore, a fraction of the variety-by-environmental condition interactions becomes part of the estimated genetic trend (Fischer et al. 2014). In summary, trends estimated from vintage trials are imprecise due to limited amount of data and also are potentially biased due to variety-by-environmental condition interactions.

The alternative is to determine trends from historical VCU trial data. These datasets are large and collected over a relatively long period of time. VCU trials are commonly organized in over-lapping cycles. Every year, a new set of genotypes enters into a two- or three-year testing cycle. If genotypes meet certain selection criteria, they are registered and become varieties at the end of a cycle. Check varieties that were registered in the past are included in all cycles as a benchmark and to connect cycles. An important aspect of VCU trial data is that it includes data on varieties as well as data on those genotypes that were tested in trials but could not make it to registration. Therefore, the population of registered varieties (post-registration population) is a subset of the population of genotypes tested in VCU trials (post-breeding population). In general, the genetic trend can be estimated for both populations. The genetic trend of interest is the genetic trend of varieties that have been registered, as only these varieties can be grown on-farm. We can therefore say that this trend is a post-registration trend. In contrast, trend estimates from VCU trial data that includes all submitted genotypes represent post-breeding trends. This may be of particular interest to breeders. Both trend estimates consider the trend for a target region and thus across the locations within the region.

For trend estimation from VCU trial data, a simple and commonly used approach is to restrict the dataset to genotypes which were either registered at the end of a cycle (i.e., varieties) or which underwent at least three or four years of testing (Mackay et al. 2011; Laidig et al. 2014, 2017; Rijk et al. 2013; Öfversten et al. 2004; de la Vega et al. 2007; Woyann et al. 2019). The idea behind this is to restrict data to the population of varieties or a population of genotypes, which is more similar to the population of interest compared to the post-breeding population of VCU trial data. Additionally, data reduction speeds up calculation. However, according to Little and Rubin’s (2002) classification of missing-data patterns, the systematic reduction of VCU trial data results in an informative missing data pattern (missing not at random; MNAR). This pattern depends on both the observed data from registered varieties and checks and on the non-used (missing but observed) data from dropped genotypes. MNAR patterns can result in biased variance component estimates if the selection of genotypes is based on the considered trait (Piepho and Möhring 2006; Hartung and Piepho 2021).

Mackay et al. (2011) were aware of this potential problem but argued that variety selection for yield is done by comparison of new genotypes against established check varieties rather than by direct selection amongst the genotypes themselves. As full data of these checks are inevitably included, data for making the selection decision are included. Moreover, the model-based adjustment for differences in cycle means mainly depends on check varieties. Again, as data from these check varieties is not reduced and thus check varieties are not subject to selection, the authors expected negligible bias if any. Similar assumptions were made in Laidig et al. (2014) and in other studies using VCU trial data. The current study aims to investigate the validity of this assumption. Simulations are set up to mimic the structure of German VCU trials including a yield-dependent selection of genotypes. Simulated data prior and after restricting it to some minimum of testing years are analysed to quantify potential bias in estimated genetic and non-genetic post-breeding trend.

## Methods

The general approach of the study was to use a real VCU trial dataset for winter wheat to estimate variance components for genetic and non-genetic sources. The structure of this dataset and the variance components were then used to simulate new datasets, including selection on the simulated trait. The real dataset and the simulated datasets were analysed with mixed models.

### VCU dataset for wheat

Winter wheat yield data from 1622 VCU trials performed by the Federal Plant Variety Office in Germany (Bundessortenamt, BSA) between 1983 and 2016 (34 years) were used (Table 1). The dataset was already used for trend estimation (Laidig et al. 2017). Each trial was performed as a split-plot design with main-plots treated or non-treated with fungicides and growth regulators. The sub-plot factor was genotype. Data used here were limited to the treated level to avoid effects resulting from the loss of tolerance or resistance of genotypes due to pathogenic adaption (Rijk et al. 2013; Laidig et al. 2014). Therefore, the design with respect to the data used reduced to a randomized complete block design (RCBD). Adjusted means for each genotype-by-trial combination at each main-plot level were available.

**Table 1:** Characteristics of the historical winter wheat dataset and the three simulated datasets

Number of	Dataset			
	Historical winter wheat data (BSA)	C	C-1	C-2
Observations	77,802	77,802	58,108	42,066
From candidates	64,792	64,792	45,293	30,233
From checks	13,010	13,010	12,815	11,833
Years	34	34	34	34
Locations	120	120	120	120
Year-by-location combinations	1302	1302	1302	1302
Trial-by-year-location combinations	1622	1622	1622	1622
Genotypes	2912	2912	1320	702
Genotype-by-year combinations	5095	5095	3491	2245
Genotype-by-location combinations	60,421	60,421	40,727	26,148
Genotype-by-year-by-location combinations	75,795	75,795	56,101	40,102

**Fig. 1:**
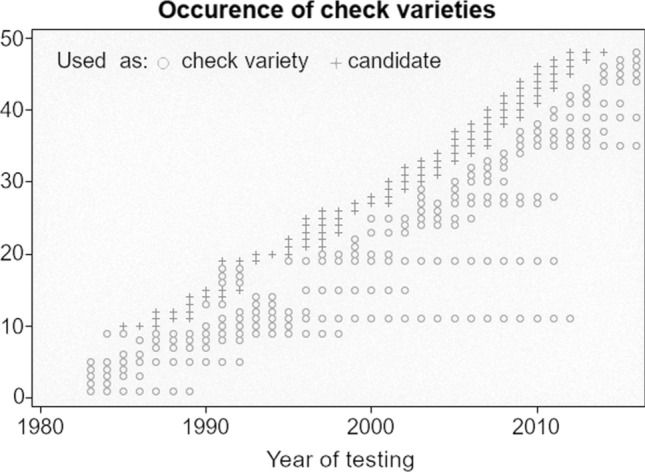
Occurrence of check varieties across years. Check varieties are numbered and shown on the y-axis. Each plus ( +) for a given year of testing means that the check variety is included as candidate genotype within the dataset, a circle (o) indicates that the check variety is included as check within the dataset. Most check varieties first occurred as candidates for three years. One or two years after registration, the registered variety was used as a check

In general, data were organized in overlapping three-year cycles. Every year a new cycle with a new set of genotypes started. One cycle consists of three trial series, i.e. three years of trials. In each year, three trial series from three subsequent cycles were tested in parallel with one series in the first, one in the second and one in the third year. All genotypes of each cycle were tested in the first year. After the first and second year on average 51% and 26% of all genotypes were culled as the aim is to register only the best genotypes as new varieties. Note that the number of genotypes tested in the first trial series increased from approximately 60 in 1983 to approximately 120 in 2016, but proportions of culled genotypes after the first series remained constant (Figure S1a). In contrast, there was an increase in the proportion of genotypes in the third year of testing from 18 to 28% (Figure S1b). The selection decision is based on empirical best linear unbiased estimates (BLUEs) of genotype means for several traits. The most important decision criterion is an index combining yield, quality and ratings of resistance (called Ertragswertzahl in German). To have a benchmark for selection and to allow comparing genotypes from different series, some well-known check varieties were included in all trials. Each check variety was included in more than one cycle and thus occurs in more than three years (Fig. 1).

The dataset comprised 2912 genotypes and had a total of 77,802 observations (Table 1). Genotypes can be divided into three groups: (1) 2901 candidates with 64,792 observations, where a candidate here means a genotype which occurs at least in its first year of testing, (2) 48 check varieties (37 were successful candidates in earlier years) that were tested as candidate or as check variety for on average 6.8 years (Fig. 1) with a total of 12,206 observations (2,001 observations as candidates), as well as (3) 190 other genotypes. All of the latter group are also included in the group of candidates. Most of the candidates were tested in two subsequent cycles for various reasons. To simplify terminology, we subsequently subsume check varieties and other genotypes under the term ‘checks’.

Only a part of the candidates was registered as varieties after testing. A total of 1592 (55%) and 618 (21%) out of 2901 candidates were culled after the first year and after the second year of testing, respectively. Furthermore, only about one half of the remaining candidates were registered as varieties later on. The data from candidates included 64,792 mean values from 64,792 genotype-by-year-by-location combinations, whereas the data of checks included 13,010 mean values from 10,003 genotype-by-year-by-location combinations. Only check varieties can occur more than once within a year-by-location combination because they can be present in different series side by side.

Within a trial series and cycle, all genotypes were tested in the same set of locations. On average, genotypes within a series were tested in 11.4, 12.6, and 24.1 locations in the first, second and third year, respectively. Within a cycle, only a few locations were repeated across years. On average 1.9, 1.3 and 7.6 locations occurred subsequently in the first two years, in the first and third and in the last two years, respectively. This resulted in a large number of tested genotype-by-location combinations (60,421) compared to the number of tested genotype-by-year-by-location combinations (75,795). The dataset is sparse in the sense that only 0.65% of the possible genotype-by-year-by-location combinations are available.

The complete dataset of historical winter wheat data is denoted as BSA. As VCU trial dataset used for trend analysis are commonly reduced by dropping genotypes tested for up to two years (Laidig et al. 2014, 2017; Piepho et al. 2014), a reduced dataset is created as well. This dataset is denoted as BSA-2. Furthermore, the data were reduced to the 48 check varieties, as the connectivity of cycles and therefore the precision of adjusting cycles mainly depends on these check varieties. This dataset is denoted as “check varieties only”.

### Analysis of VCU data

The complete and reduced historical datasets were analysed for the trait yield using a three-way model with factors year, location and genotype. The model is

1 $$\overline{y}_{ijkl} \, = \,\mu \, + \,Y_{k} \, + \,L_{j} \, + \,G_{i} \, + \,\left( {LY} \right)_{jk} \, + \,\left( {LYT} \right)_{jkl} \, + \,\left( {GL} \right)_{ij} \, + \,\left( {GY} \right)_{ik} \, + \,\left( {GLY} \right)_{ijk} \, + \,e_{ijkl}$$

where $\overline{y}_{ijkl}$ is the mean of genotype *i* in trial *l* of year *k* and location *j* (Piepho and Michel 2000). For all candidates there is only a single mean within a year-by-location combination. Only check varieties can occur twice or three times within a year-by-location combination. This is the case if two or three series were tested in the same year and location. Thus, the separation between $\left( {GLY} \right)_{ijk}$ and the error $e_{ijkl}$ is based on check varieties only. The term $\mu$ is the intercept, $Y_{k}$ is the *k*th year effect, $L_{j}$ is the *j*th location effect and $G_{i}$ is the *i*th genotype effect. The effects $\left( {LY} \right)_{jk}$, $\left( {GL} \right)_{ij}$, $\left( {GY} \right)_{ik}$, and $\left( {GLY} \right)_{ijk}$ are the interaction effects of the corresponding main effects, and $\left( {LYT} \right)_{jkl}$ is the trial effect within a year-by-location combination. Again, separation of trial effects and location-by-year effects is based on check varieties only. All effects (except the intercept) were assumed as random. Homogeneous variances were assumed for all effects. Following the approach of Piepho et al. (2014), the model was extended by replacing genotype and year main effects as follows:

$$G_{i} \, = \,\beta \cdot r_{i} \, + \,H_{i}\,\,\,{\rm{and}} \,\,Y_{k} \, = \,\gamma \cdot t_{k} \, + \,Z_{k}$$

where $t_{k}$ is a numeric variable for the year of testing and $r_{i}$ is a numeric variable for the year of first testing, $\beta$ and $\gamma$ are the corresponding slopes, and $H_{i}$ and $Z_{k}$ are the corresponding random deviations of $G_{i}$ and $Y_{k}$ from the corresponding regression line. The slope $\beta$ represents the genetic trend while the slope $\gamma$ represents the non-genetic trend. The complete model can be described as:

2 $$\overline{y}_{ijkl} \, = \,\mu \, + \,\gamma t_{k} \, + \,Z_{k} \, + \,L_{j} \, + \,\beta r_{i} \, + \,H_{i} \, + \,\left( {LY} \right)_{jk} \, + \,\left( {LYT} \right)_{jkl} \, + \,\left( {GL} \right)_{ij} \, + \,\left( {GY} \right)_{ik} \, + \,\left( {GLY} \right)_{ijk} \, + \,e_{ijkl}$$

This model differs from the models used in Laidig et al. (2014, 2017), Mackay et al. (2011), Öfversen et al. (2004) and Piepho et al. (2014) in two ways: (1) it includes a trial effect and (2) it separates error and three-way interaction effects. In both cases the separation is based on check varieties. The historical dataset was analysed using PROC HPMIXED from the SAS system. To avoid memory problems, additional factors were defined for all interaction effects. These factors have as many levels as there are interaction effects. The numeric variables year of testing and year of first testing were not centered.

To check whether the selection intensity of the BSA has changed during the study period, the proportion of selected genotypes among the tested genotypes in the first year was regressed on the first year of testing by fitting a logistic regression. The linear predictor corresponds to the expected value of a simple linear regression. The analysis assumed a binomial distribution with the logit link function.

### Data simulation

The main purpose of the data simulation was to mimic the historical VCU trial datasets, including the selection exercised by the testing authorities. Data were simulated according to the model used for analyzing VCU trial dataset (model 2). All effects were simulated as normally distributed with zero expectation and independent and identically distributed effects. VCs estimated from the historical data (Table 2) were used to simulate new datasets. Additionally, a second set of VC was used, which was identical to the first set but had the genotype-by-year variance increased from 3.16 to 55.75. In this case, the genotype-by-year variance was 4.2 times larger than the genotype variance, while in historical data, the genotype variance is 4.2 times larger than the genotype-by-year variance. In contrast to results of VCU trial dataset analysis, zero slopes for post-breeding non-genetic and genetic trend were simulated. This entails no loss of generality, as we are only interested in assessing bias and empirical best linear unbiased estimation (BLUE) is generally unbiased (Searle et al. 1992). Simulated datasets were designed to mimic the historical VCU trail dataset reproducing the exact same structure. This means that the simulated data mimicked the historical data in the sense that the same number of genotypes were tested in the same number of years and locations and that there was always a pair of genotypes (one genotype in the historical dataset and one in the simulated dataset) with the same amount of testing in the same years and locations. This required that the same number of candidates were simulated in each year and series as in the historical data. As in the historical data only candidates were culled during testing, the whole dataset to be simulated was split into 64,792 observations from candidates and 13,010 observations from checks. Data for candidates were split into cycles and the complete data for three years was simulated for each cycle. Afterwards, data from the first year of each cycle was used to select candidates tested in the corresponding second year. Candidates tested in the third year of each cycle were selected from these candidates using data of the corresponding first and second year of testing. The proportion of candidates tested in the second and third year of each cycle corresponds to the proportion tested in the cycles of the historical data. Technically, this required exchanging genotype and genotype-by-location labels after each selection step to make sure that data of the selected candidates are indeed occurring in the successive year in the historical dataset. Genotype effects and genotype-by-location effects of candidates which were used as check varieties later on, were transferred to the simulated values of these check varieties. Data for all other checks were simulated using model (2) and not changed afterwards. Year, location, year-by-location and trial-by-year-location effects were simulated once per simulation run and used for all candidates in all cycles and all checks.

**Table 2:** Variance component estimates absolute and relative in parenthesis as well as trend estimates with their standard error given in parenthesis for a series of 1622 winter wheat trials across 34 years using all genotypes available (BSA) and dropping genotypes with up to two years of testing (BSA-2)

Parameter	Estimate for dataset in dt^2^ ha^−2^		
	BSA	BSA-2	Check varieties only
*Variance component*			
Year	25.13 (13%)	23.89	22.99
Location	53.15 (27%)	53.50	54.08
Year-by-location	74.14 (37%)	73.98	72.90
Year-by-location-by-trial	8.83 (4%)	9.03	9.55
Genotype	13.27 (7%)	12.10	11.17
Genotype-by-year	3.16 (2%)	2.94	4.30
Genotype-by-location	2.25 (1%)	2.16	2.38
Genotype-by-year-by-location	9.62 (5%)	9.01	9.19
Error	9.36 (5%)	9.41	9.17

Trend	Estimate (standard error) for dataset in dt ha^−1^ year^−1^		
Genetic	0.559 (0.029)	0.553 (0.031)	0.555 (0.050)
Non-genetic	0.174 (0.100)	0.126 (0.098)	0.153 (0.099)

Separate selection steps were performed for each cycle. In each cycle, the selection of candidates in the first year was based on the following model

3 $$\overline{y}_{ij} \, = \,\mu \, + \,L_{j} \, + \,G_{i} \, + \,e_{ij}$$

where $\mu$ is the confounded effect of the intercept and the main effect of the considered year, $L_{j}$ is the confounded main effect of the *j*th location, the interaction effect of the *j*th location in the considered year and the *l*th trial effect within the considered location and year. Similarly, $G_{i}$ is the confounded main effect of the *i*th genotype with the interaction effect of genotype-by-year in the considered year. The error effect $e_{ij}$ denotes the confounded effect of genotype-by-location interaction, genotype-by-location-by-year interaction and error effect. All effects were assumed to be random except the intercept. Candidates were selected based on their best linear unbiased predictions (BLUP) for $G_{i}$. Thus, selection in the simulation only depends on the single trait yield. Furthermore, BLUPs were used (in contrast to BLUEs as was used in the historical VCU trials), as the former minimize the mean squared error (Robinson 1991; Piepho et al. 2008). In each cycle, the selection of candidates in the second year was based on the following model:

4 $$\overline{y}_{ijk\,} \, = \,\,\mu \,\, + \,\,Y_{k} \, + \,\,L_{j} \,\, + \,\,G_{i} \,\, + \,\,\left( {LY} \right)_{jk} \, + \,\,\left( {GL} \right)_{ij} \, + \,\left( {GY} \right)_{ik} \, + \,\left( {GLY} \right)_{ijk}$$

where $\left( {GLY} \right)_{ijk}$ now is the confounded effect of three-way interaction and error effect, $\left( {LY} \right)_{jk}$ is the confounded effect of the year-by-location interaction effect and the trial effect of this year-by-location combination. A separation of both effects is not possible as the data included a single trial per year-by-location combination only. A separation of these effects requires a joint analysis of more than one cycle at a time. Model (4) is in line with the across-cycle models used in Laidig et al. (2014, 2017), Mackay et al. (2011), Öfversten (2004), Piepho et al. (2014) and de la Vega et al. (2007). It differs somewhat from the models and methods actually used for selection in the German historical VCU trials, where selection is based on model (3) and second year data only. The selection therefore differed in three ways from selection performed by the Federal Plant Variety Office in Germany: First, multi-trait selection was replaced by single-trait selection. Second, BLUE for $G_{i}$ was replaced by best linear unbiased prediction (BLUP) for $G_{i}$ and third, all available data within a cycle was used for yearly selection, whereas in VCU trials, selection is based on the current year’s data only. All of these three changes should increase selection intensity in the simulated data compared to the actual VCU data. Adding candidates of all simulated cycles and the checks forms the complete dataset.

A total of four simulations were performed. Three datasets were created within each run of each of the four simulations. For the first three simulations, variance components estimated from the historical data (BSA; Table 2) were used to simulate new datasets.

The simulated datasets in the first simulation that mimicked the VCU trial dataset were denoted as C (for complete). Additionally, C datasets were modified afterwards by dropping some observations. Specifically, datasets C-1 and C-2 were created by dropping candidates that were tested only in one or in up to two years. Dropping candidates which were tested for one or up to two years results in an informative missing data pattern (Little and Rubin 2002; Piepho and Möhring 2006). The three simulated datasets (C, C-1 = C \{candidates tested for one year}, and C-2 = C \ {candidates tested for less than three years}) were created in each of 500 simulation runs. They share the years, locations and trials but vary in the number of genotypes tested (Table 1). Datasets C and BSA as well as C-2 and BSA-2 have the same size, respectively.

In the second simulation, the historical VCU trial dataset was reduced by randomly dropping duplicates in genotype-by-year-by-location combinations. The drop-out mechanism here is completely at random (MCAR) and thus should not affect expectation of parameters estimated from the dataset. Duplicates only occurred for check varieties. As a consequence, the data had a single mean for each genotype-by-year-by-location combination within the dataset. Therefore, model (2) simplifies to model (5) given below. Thus, the model for analysis is identical to the model for simulating the data. After dropping duplicates, data were simulated analogously to dataset C. The simulated dataset was denoted as SM (for single mean). It was reduced to SM-1 and SM-2 by dropping genotypes tested for up to one or up to two years. The datasets SM, SM-1, and SM-2 had 75,795, 55,106 and 41,102 observations, respectively. As the additional drop-out of check varieties’ data was completely at random (MCAR), this step did not change the missing data pattern. Thus, the datasets C and SM had a missing-at-random (MAR) data pattern. The other four datasets (C-1, C-2, SM-1 and SM-2) had an informative missing pattern (MNAR). 100 simulation runs were performed for the second simulation.

The third simulation was similar to the first one, but replaced the yield-based selection by a random selection. In this case, the missing data pattern is MCAR. This simulation served as a benchmark for the other simulations.

In the fourth simulation, the genotype-by-year variance was increased to 55.75. This fourth simulation was added to check if bias in trends can be modified when increasing genotype-by-year variance. All remaining simulation steps including the number of simulation runs are identical to the first simulation. Datasets were denoted as I, I-1 and I-2 (for the increase in genotype-by-year VC).

### Analysis of simulated datasets

All datasets were analysed using the same model. As model (2) is computationally demanding, it was not used within the simulation. The model was reduced to

5 $$\overline{y}_{ijkl} \, = \,\mu \, + \,\gamma t_{k} \, + \,Z_{k} \, + \,L_{j} \, + \,\beta r_{i} \, + \,H_{i} \, + \,\left( {LY} \right)_{jk} \, + \,\left( {LYT} \right)_{jkl} \, + \,\left( {GL} \right)_{ij} \, + \,\left( {GY} \right)_{ik} \, + \,e_{ijkl}$$

where $e_{{{\text{ijkl}}}}$ now is the confounded effect of the three-way interaction $\left( {GLY} \right)_{ijk}$ and the error in (2). This simplification is in line with most papers analyzing VCU trial data for trend analysis (Laidig et al. 2014; Laidig et al. 2017; Mackay et al. 2011; Öfversten 2004; Piepho et al. 2014; de la Vega 2007). Note that model (5) accounts for trial main effects that are commonly ignored, but ignores the covariance of multiple observations on the same genotype (check) in the same year-by-location combination due to an identical genotype-by-year-by-location effect ($\left( {GLY} \right)_{ijk}$). This covariance only occurs in datasets C, C-1 and C-2. For these datasets, the model for analysis (ignoring covariance) differs from the model for data simulation (modelling covariance). Thus, the comparison between C and SM datasets allows quantifying the bias resulting from this simplification. The comparison between results from the first and third simulation allows evaluating the effect of the size of genotype-by-year variance on the bias in trend estimation. The simulation of datasets and their analyses were performed with SAS.

### Evaluation criteria

In each simulation run and each dataset, the variance components for all effects were estimated. Subsequently, these estimates were averaged across simulation runs. Furthermore, the slopes for genetic and non-genetic trends were estimated. The mean squared error (MSE) between estimated and simulated values of $H_{i}$ of all genotypes (including checks) tested in at least three years were calculated. Note that all values simulated for the post-breeding population, thus evaluation criteria refers to the post-breeding population. A weighted average was then computed from MSE values using a meta-analytic approach with weights corresponding to the inverse squared standard errors (Borenstein et al. 2009). Additionally, the rank correlation was calculated between estimated and simulated values of $H_{i}$ of all genotypes (including checks) tested in at least three years. Correlations were averaged across simulation runs.

## Results

### Results from historical VCU trial dataset

The analysis of the historical VCU trial datasets showed large variances for year, location and year-by-location compared to genetic variances (Table 2). This is in line with, e.g. Laidig et al. (2008). There is a strong positive genetic trend and a small but positive non-genetic trend. The 95% confidence interval for the genetic trend do not include zero.

Genetic VCs (genotype, genotype-by-year, genotype-by-location, genotype-by-year-by-location) were slightly smaller for BSA-2 compared to BSA. Additionally, the estimated non-genetic trend was smaller for BSA-2 compared to BSA. If data were reduced to the 48 check varieties only, VC and trend estimates were comparable to the estimates obtained for the complete data.

### Results from simulated datasets

For SM, estimated 95% confidence interval for all VCs covered the simulated value and no bias was detected. In contrast, analysis of C, C-1, C-2, SM-1, and SM-2 showed biased VC estimates compared to the VC simulated (Tables 3, 4). For C, C-1 and C-2, the genotype-by-location VCs were over-estimated and the confounded genotype-by-year-by-location and error VCs as well as the year-by-location-by-trial VC were underestimated. For C, the genotype-by-year VC estimate was slightly larger than simulated. For C-1 and C-2, genotype and genotype-by-year VCs were underestimated. Both VC were underestimated in SM-1 and SM-2 as well. The genotype and genotype-by-year VC estimates differed between datasets C, C-1, C-2, SM, SM-1, and SM-2, with larger VC in C and SM compared to datasets C-1, C-2 and SM-1 and SM-2, respectively. Differences got larger in C-2 and SM-2 compared to C-1 and SM-1, respectively (Tables 3, 4). All other 95% confidence intervals of VC estimates covered the true values (Table 3, 4).

**Table 3:** Simulated post-breeding and average estimated variance component (VC) values as well as simulated post-breeding and estimated trends, the mean squared error (MSE) of estimated genotype best linear unbiased predictions (BLUPs) for simulated datasets C, C-1 and C-2 across 500 simulations

Parameter	Values used for simulation (BSA)	Estimate for dataset (95% confidence interval)		
		C	C-1	C-2
*Variance component*				
Year	25.13	25.18 (24.57; 25.78)	25.22 (24.61; 25.83)	25.21 (24.60; 25.82)
Location	53.15	53.60 (52.82; 54.37)	53.66 (52.88; 54.44)	53.65 (52.87; 54.43)
Year-by-location	74.14	74.20 (73.89; 74.51)	74.18 (73.87; 74.49)	74.25 (73.94; 74.57)
Year-by-location-by-trial	8.83	**8.74** (8.68; 8.81)	8.79 (8.72; 8.86)	**8.71** (8.64; 8.78)
Genotype	13.27	13.26 (13.23; 13.30)	**6.52** (6.48; 6.55)	**5.90** (5.86; 5.94)
Genotype-by-year	3.16	**3.18** (3.16; 3.19)	**3.01** (3.00; 3.02)	**3.11** (3.09; 3.12)
Genotype-by-location	2.25	**2.51** (2.50; 2.52)	**2.53** (2.51; 2.54)	**2.53** (2.51; 2.54)
Genotype-by-year-by-location	9.62	**18.69** (18.67; 18.70)	**18.66** (18.65; 18.68)	**18.65** (18.64; 18.67)
Error	9.36			
*Trend*				
Genetic	0	0.000 (− 0.002; 0.003)	**0.086** (0.084; 0.089)	**0.115** (0.113; 0.118)
Non-genetic	0	− 0.000 (− 0.009; 0.009)	− **0.070** (− 0.079; − 0.061)	− **0.106** (− 0.115; − 0.097)
*Evaluation criterion*				
MSE^§^	–	1.42 (1.41; 1.43)	8.97 (8.94; 9.01)	16.79 (16.74; 16.84)

The dataset C mimics the complete historical VCU dataset. The datasets C-1 and C-2 were created from C by dropping genotypes tested up to one or two years, respectively. Values printed in bold have a confidence interval that does not include the value used for simulation. Values in parenthesis represent the 95% confidence interval. Values are given in dt^2^ ha^−2^ for VC and MSE and dt ha^−1^ year^−1^ for trend estimates

^§^Mean squared error between genotype BLUPs and simulated true values

**Table 4:** Simulated post-breeding and average estimated variance component (VC) values as well as simulated post-breeding and estimated trends, the mean squared error (MSE) of estimated genotype best linear unbiased predictions (BLUPs) for simulated datasets SM, SM-1 and SM-2 across 100 simulations

Parameter	Values used for simulation (BSA)	Estimate for dataset (95% confidence interval)		
		SM	SM-1	SM-2
*Variance component*				
Year	25.13	25.19 (23.88; 26.49)	25.13 (23.94; 26.59)	25.23 (23.91; 26.55)
Location	53.15	52.87 (51.16; 54.58)	52.94 (51.22; 54.65)	52.90 (51.18; 54.61)
Year-by-location	74.14	73.78 (73.17; 74.39)	73.73 (73.11;74.35)	73.78 (73.16; 74.40)
Year-by-location-by-trial	8.83	8.83 (8.70; 8.97)	8.92 (8.79; 9.06)	8.85 (8.71; 8.98)
Genotype	13.27	13.33 (13.23; 13.44)	**6.48** (6.40; 6.56)	**5.88** (5.79; 5.97)
Genotype-by-year	3.16	3.15 (3.12; 3.19)	**2.99** (2.96 ;3.02)	**3.08** (3.04; 3.11)
Genotype-by-location	2.25	2.25 (2.22; 2.28)	2.26 (2.23; 2.29)	2.27 (2.24; 2.30)
Genotype-by-year-by-location	9.62	18.97 (18.94; 19.00)	18.94 (18.91; 18.97)	18.94 (18.91; 18.98)
Error	9.36			
*Trend*				
Genetic	0	− 0.002 (− 0.007; 0.004)	**0.086** (0.080; 0.091)	**0.114** (0.108; 0.119)
Non-genetic	0	− 0.002 (− 0.022; 0.017)	− **0.073** (− 0.092; − 0.054)	− **0.108** (− 0.127; − 0.089)
*Evaluation criterion*				
MSE^§^	–	1.43 (1.41; 1.45)	9.12 (9.07; 9.17)	17.00 (16.93; 17.08)

The datasets SM-1 and SM-2 were created from SM by dropping genotypes tested up to one or two years, respectively. Values printed in bold have a confidence interval that does not include the value used for simulation. Values in parenthesis represent the 95% confidence interval. Values are given in dt^2^ ha^−2^ for VC and MSE and dt ha^−1^ year^−1^ for trend estimates

^§^Mean squared error between genotype BLUPs and simulated true values

Means of estimated genetic and non-genetic trends are close to zero with 95% confidence interval covering the value zero for C and SM (Tables 3, 4). In contrast, a positive genetic trend and a negative non-genetic trend can be observed for C-1, C-2, SM-1 and SM-2. Trends in C-1 and SM-1 as well as in C-2 and SM-2 were similar with larger deviations from zero in datasets dropping genotypes tested for up to two years. In all cases, zero is not contained in the 95% confidence interval (Tables 3, 4). Estimated trends became larger when increasing the genotype-by-year variance (Table S1). Furthermore, dropping data from genotypes tested only one or two years resulted in an increased MSE. If yield based selection was replaced by random selection, no differences between complete and reduced data can be detected. In this case, trend estimates were close to zero (results not shown). Rank correlation of genotype BLUPs and true simulated values were similar for all datasets (0.86–0.87 with slightly larger values for C and SM, respectively).

## Discussion

The simulations showed that when using a proper mixed model and all available data (model 3 with datasets SM), VC estimates were unbiased. Additionally, non-genetic trend and post-breeding genetic trend can be estimated without bias. This is expected as datasets are then large with an MAR data pattern and the analysis is based on restricted maximum likelihood estimation (Piepho and Möhring 2006; Hartung and Piepho 2021). Similar results were observed, if the yield-based selection was replaced by a random selection. Again, the missing data pattern is MAR. It should be stressed here that we would expect similar results for all datasets (including those with MAR data pattern) when accounting for the relationship between genotypes e.g. via pedigree or marker-based relationship matrices (van der Werf and de Boer 1990), but note that such information is not usually available for VCU trials, and was not available here.

For the other datasets, the missing data pattern is NMAR. In this case, simulation showed that there is a bias in VC estimates and trend estimates. It is important to re-iterate that our assessment of bias in VC and trend estimates relates to the post-breeding population and thus to the population entering the VCU trials. In both the historical VCU trial data and our simulation, the population of registered varieties (post-registration) differs from the population of genotypes submitted by breeders for registration (post-breeding). Submitted genotypes in the post-breeding population are the result of breeders’ selection. In contrast, registered varieties are based jointly on selection by breeders in their trials and selection exercised by examination offices in VCU trials. The post-registration population is a sub-population selected from the post-breeding population. Therefore, within a cycle both populations differ systematically. Selection performed in VCU trials causes selection gain and therefore results in larger expected means in the post-registration population compared to the corresponding post-breeding population.

For the datasets C-1, C-2, SM-1, SM-2, I-1 and I-2, the simulation showed that there are two sources of bias for VC estimates: the differences between model (2) used for data simulation and model (5) used for analysis, as well as the informative drop-out in these reduced datasets.

Bias in VC estimates found in C, C-1 or C-2, but not in the corresponding datasets SM, SM-1 or SM-2, were caused by the simplification made in model (5). In Germany, VCU trials are organized in overlapping series. In each year, a new series with new genotypes but usually the same check varieties is started. As these series are tested in the same locations in two or three years, check varieties can occur more than once in a given year-by-location combination. This results in a positive correlation between these mean values. The common approach (model 3) is to ignore this correlation (Piepho et al. 2014). In our simulations, ignoring the correlation resulted in slightly biased VC estimates for year-by-location-by-trial, genotype-by-location and the confounded variance of genotype-by-year and error effects. Trend estimates were unaffected.

Bias seen in VC estimates from reduced datasets only were caused by the informative drop-out. The bias in the genotype VC is based on selection of the best-performing genotypes (Piepho and Möhring 2006). It is a bias relative to the variance simulated for the post-breeding population. The variance of the post-registration population is expected to be smaller compared to the post-breeding population as poorly performing genotypes are dropped (Schüler et al. 2001). Therefore, it is not clear whether the post-registration genotype VC is estimated with bias. The bias seen for the post-breeding genotype-by-year VC is based on selection too, as in the first year of testing the genotype and genotype-by-year effects are confounded. Again, it remains unclear whether there is bias of the VC estimates in relation to the post-registration population.

For all reduced datasets with informative missing data pattern, genetic post-breeding trend is overestimated and agronomic trend is underestimated. Bias disappeared when using random selection and increased with increasing genotype-by-year VC. Trends are estimated by comparing means of subsequent cycles. To adjust means of different cycles, data of check varieties are required. In VCU trial data from Germany, about 15% of data belongs to check varieties. As stated above, means differ systematically between post-breeding and post-registration populations. However, if the selection gain reached by examination offices is constant for all cycles, the trend estimated from both populations are expected to be similar. They can vary in case of a temporally varying selection intensity within VCU trials. In both the German historical VCU trial data and our simulated data, the proportion of genotypes tested in the third year increased with time (Figure S1b). This increase should decrease the selection gain reached by examination offices in the third year. As the gain decreases in more recent cycles, this should reduce post-registration genetic trend estimate compared to post-breeding genetic trend estimates. In contrast, our simulation showed overestimated genetic trends when using data from candidates with at least two or three years of testing. Therefore, the observed bias in post-breeding trend estimates probably is based on the selection of candidates. Candidates were selected according to their estimated yield in the current year (current and previous years in the second selection step), and thus on the confounded effects of genotype and genotype-by-year. Therefore, candidates with positive genotype-by-year effects are more likely selected for further testing compared to candidates with negative genotype-by-year effects. As this positive effect cannot be reproduced in subsequent years, it is covered by the regression on the year of testing and thus is interpreted as negative non-genetic trend. Furthermore, as the simulated overall trend is zero, the estimated genetic trend must be positive.

The simulation results provide an upper bound for the bias for post-breeding genetic and non-genetic trend expected from informative drop-out, as selection in our simulation was based on BLUPs of a single trait only, whereas in actuality selection decisions are more complex. Indeed, the selection of the BSA are based on BLUEs for yield, as well as for a large number of quality and resistance traits. If selection is based on more traits, the selection intensity for yield is much lower than assumed in our simulation. Note that we replaced selection based on BLUE by BLUP-based selection, as BLUPs have smaller mean squared error even in small experiments (Forkman and Piepho 2013). BLUP minimizes the mean squared error and thus maximizes the correlation of estimated and true ranking of the genotypes (Searle et al. 1992). In both cases, selection decisions probably get better and selection intensity is increased. Additionally, we replaced single-year selection after the second year by a selection based on both testing years. Thus, selection in the second year prefers candidates with a positive genotype-by-year effect in both years. As selection intensity in our simulation is larger than in the historical VCU trials, the observed bias seen in our simulation is larger too. For this reason, bias in trend estimation from data-reduced historical VCU trial analysis is expected to be smaller compared to our simulation. The genotype VC estimates in BSA-2 and C-2 or SM-2 gave some idea about the strength of yield-dependent selection in historical VCU data. In our simulations, the genotype variance was reduced to less than 50% compared to the post-breeding VC, whereas the genotype VC estimate in BSA-2 was still larger than 90% of the estimate in BSA. Nevertheless, an informative missing data pattern has the risk of biased trend estimates.

Trends seen in VCU trial datasets are based to genetic and non-genetic sources. To dissect these, several approaches can be used. If weather data is available, a crop growth model can be used to correct for yield differences due to systematic changes in climate (Gonzalo et al. 2022; Rizzo et al. 2022; Hadasch et al. 2020). This allows separating climate and non-climate sources of trends. Piepho et al. (2014) dissected genetic and non-genetic trends by adding two linear regressions in their mixed model, one for the year of first testing (defined as genetic trend) and one for the calendar year of testing (defined as non-genetic trend). While the approach is easy to apply, it heavily depends on the connectivity of the data and thus on the occurrence of check varieties across and within years. Adding data from vintage trials can extend the connectivity of the data. The current paper does not extend the database but only uses historical data, as this is the common approach when analyzing VCU datasets. Additionally, weather data were not available, thus the current paper used the mixed model approach of Piepho et al. (2014) adding only linear regressions for genetic and non-genetic trends. The assumption of linearity was not tested formally with the current data, though regression plots suggest no grave departures from linearity. Beche et al. (2014), Grassini et al. (2013) and Fischer et al. (2014) suggested to use a linear time trend to describe current and future change rates for yield (Fischer 2015). In contrast to this, Calderini and Slafer (1998) and Öfversten et al. (2004) found non-linear trends. Boken (2000) and Finger (2010) show that quadratic models fitted the trend better than the linear model, revealing a significant inflexion point in the trend. Slafer and Peltonen-Sainio (2001) stated that on-farm yield seemed to stagnate for some crops in developed countries during the last years. Such a plateau can be fitted by broken-stick models (Calderini and Slafer 1998). Another alternative is a linear regression for logarithmically transformed data (Öfversten et al. 2004). This corresponds to a constant relative change over time. Again, the deviations from linearity of the observed trend across all years was not checked in the current study.

The current study is based on a mixed model analysis due to its easy application. In general, other approaches are possible. As the dataset is large and thus there is a lot of information on VC estimates and variety means, we do not expect relevant differences when using a Bayesian approach. As we are interested in the interpretation of slope estimates for two specific time covariates only, black-box approaches such as neural networks and other machine learning approaches provide no advantages, and these methods make it difficult to account for the year, location and genotype main effects and interaction. Thus, mixed models are the method of choice for the problem at hand.

The estimated post-breeding genetic trend from the considered winter wheat data across all quality groups was 0.56 dt ha^−1^ year^−1^ as reported in Laidig et al. (2017) for the same trials and the same period. Cormier et al. (2013) reported a similar trend of 0.51 dt ha^−1^ year^−1^ in France. Lower genetic gains were estimated in Hartl et al. (2011) and Bilgin et al. (2015). For a detailed discussion of potential reasons for differences in genetic gain estimates see Laidig et al. (2017). Rizzo et al. (2022) reported much lower estimates of genetic gain based on on-farm data, explaining most of the visible trend by climatic variables. The latter study used different methods to predict genetic gain, calculating the genetic trend from three independent models using three datasets, one for climatic trend, one for technological trend and one for total trend. We believe that the approach to calculate the genetic trend as differences of trend estimates from three independent linear models leads to a downward bias in case that predictor variables interact or are positively correlated, hence likely leading to underestimating genetic trend. In all other studies mentioned above, the estimated trend is large compared to the upper bound of bias (0.11 dt ha^−1^ year^−1^) shown in our simulation when dropping genotypes tested for up to two years. Note that bias for historical VCU trial data can vary between crops as the ratio of genotype to genotype-by-year variance may differ from the ratio found in winter wheat. For smaller ratio and lower selection intensity the bias is expected to be smaller than the one seen in our simulation. At the same time, reduction of data reduced computational burden, so that there could be reasons to accept the bias of in trend estimates from reduction of VCU trial data. An unbiased estimation of post-registration trends is not possible from VCU trial data. Unbiased post-registration trends require data obtained after registration. In Germany, data of post-registration trials of registered varieties, performed by federal states in Germany (Landessortenversuche), can be used for post-registration trend estimation.

## Supplementary Information

Below is the link to the electronic supplementary material.

Supplementary file1 (DOCX 58 kb)

## Data Availability

The authors declare that the program code is available as supplemental material.

## Abbreviations

BLUE: Best linear unbiased estimation
BLUP: Best linear unbiased prediction
BSA: Bundessortenamt Federal Plant Variety Office in Germany
MAR: Missing at random
MCAR: Missing completely at random
MNAR: Missing not at random
MSE: Mean squared error
VC: Variance component
VCU: Value for cultivation and use
