# Supplementary material for: Effects of systematic data reduction on trend estimation from German registration trials
Source: Theor Appl Genet. 2023 Jan 23;136(1):21. doi: 10.1007/s00122-023-04266-5 (PMC9870826; doi:10.1007/s00122-023-04266-5)
Supplement: Supplementary file 1 — Supplementary file1 (DOCX 58 kb) [file 122_2023_4266_MOESM1_ESM.docx]

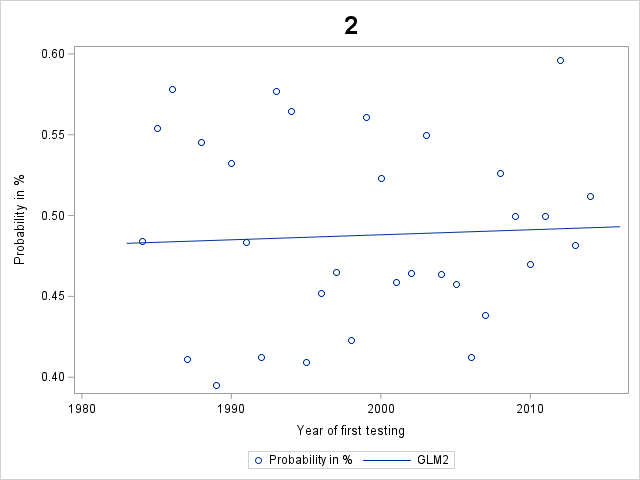

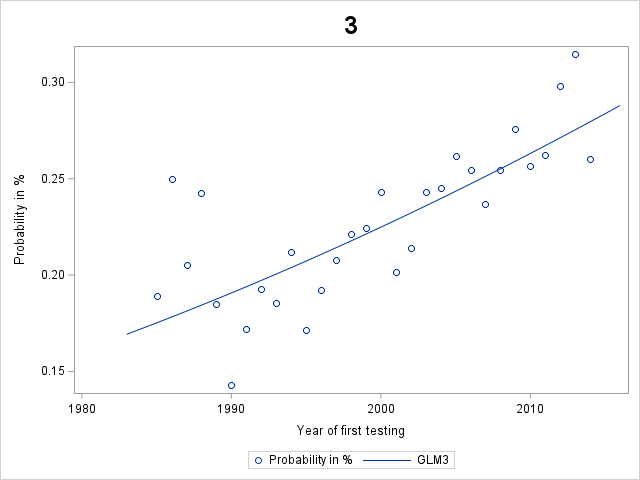


Figure S1: Probability that a genotype tested in the first year is tested in the (a) second and (b) third year. A logistic regression was fitted in (a) with $logit\left( p_{year} \right)=-2.54+0.0012\cdot year$, the slope was non-significant (p=0.7681) and (b) with $logit\left( p_{year} \right)=-42.80+0.0208\cdot year$, the slope was significant (p=0.0005).

Table S1: Simulated and average estimated variance component (VC) values as well as simulated, estimated trends, the mean squared error (MSE) of estimated genotype best linear unbiased predictions (BLUPs) for simulated datasets I, I-1 and I-2 across 500 simulations.

| Parameter | Values used for simulation | Estimate for dataset (95% confidence interval) | | |
| --- | --- | --- | --- | --- |
|  |  | I | I-1 | I-2 |
| *Variance component* |  |  |  |  |
| Year | 25.13 | 25.19 (24.57;25.81) | 25.45 (24.81;26.06) | 25.50 (24.85;26.14) |
| Location | 53.15 | 53.59 (52.81;54.36) | 53.74 (52.96;54.52) | 53.67 (52.89;54.44) |
| Year-by-location | 74.14 | 74.20 (73.89;74.51) | 73.73 (73.83;74.45) | 74.28 (73.96;74.59) |
| Year-by-location-by-trial | 8.83 | **8.73** (8.66;8.79) | 8.83 (8.76;8.90) | **8.70** (8.63;8.76) |
| Genotype | 13.27 | 13.38 (13.23;13.30) | **5.31** (5.21;5.40) | **5.41** (5.30;5.52) |
| Genotype-by-year | 55.75 | 55.67 (55.52;55.82) | **52.39** (52.24;52.53) | **52.01** (51.84;52.19) |
| Genotype-by-location | 2.25 | **2.52** (2.51;2.53) | **2.56** (2.54;2.57) | **2.54** (2.52;2.55) |
| Genotype-by-year-by-location | 9.62 | **18.68** (18.67;18.70) | **18.55** (18.63;18.66) | **18.65** (18.63;18.67) |
| Error | 9.36 |  |  |  |
| *Trend* |  |  |  |  |
| Genetic | 0 | 0.002 (-0.005;0.009) | **0.525** (0.518;0.532) | **0.640** (0.633;0.647) |
| Non-genetic | 0 | -0.000 (-0.011;0.010) | **-0.531** (-0.542;-0.521) | **-0.667** (-0.678;-0.657) |
| *Evaluation criterion* |  |  |  |  |
| MSE§ | - | 7.51 (6.71;8.31) | 11.87 (10.62;13.12) | 13.46 (12.08;14.84) |

Note. The datasets I-1 and I-2 were created from I by dropping genotypes tested up to one or two years, respectively. Values printed in bold have a confidence interval that does not include the value used for simulation. Values in parenthesis represent the 95% confidence interval. Values are given in dt^2^ ha^-2^ for VC and MSE and dt ha^-1^ year^-1^ for trend estimates.

§ Mean squared error between genotype BLUPs and simulated true values.
